# The transcriptome of MHV‐infected RAW264.7 cells offers an alternative model for macrophage innate immunity research

**DOI:** 10.1002/ame2.12443

**Published:** 2024-07-11

**Authors:** Yun Liu, Ting‐Ting Feng, Wei Tong, Zhi Guo, Xia Li, Qi Kong, Zhi‐Guang Xiang

**Affiliations:** ^1^ Institute of Laboratory Animal Sciences, Chinese Academy of Medical Sciences (CAMS) and Comparative Medicine Center Peking Union Medical College (PUMC) Beijing P.R. China

**Keywords:** coronavirus, innate immunity, macrophage, transcriptome

## Abstract

**Background:**

Macrophages are the primary innate immune cells encountered by the invading coronaviruses, and their abilities to initiate inflammatory reactions, to maintain the immunity homeostasis by differential polarization, to train the innate immune system by epigenic modification have been reported in laboratory animal research.

**Methods:**

In the current in vitro research, murine macrophage RAW 264.7 cell were infected by mouse hepatitis virus, a coronavirus existed in mouse. At 3‐, 6‐, 12‐, 24‐, and 48‐h post infection (*hpi.*), the attached cells were washed with PBS and harvested in Trizol reagent. Then The harvest is subjected to transcriptome sequencing.

**Results:**

The transcriptome analysis showed the immediate (3 *hpi.*) up regulation of DEGs related to inflammation, like *Il1b* and *Il6*. DEGs related to M2 differential polarization, like *Irf4* showed up regulation at 24 *hpi*., the late term after viral infection. In addition, DEGs related to metabolism and histone modification, like *Ezh2* were detected, which might correlate with the trained immunity of macrophages.

**Conclusions:**

The current in vitro viral infection study showed the key innated immunity character of macrophages, which suggested the replacement value of viral infection cells model, to reduce the animal usage in preclinical research.

## Graphical abstract

The macrophages were infected with mouse hepatitis virus (MHV). The transcriptome analysis showed the immediate up regulation of different expressed genes (DEGs) related to inflammation. DEGs related to M2 differential polarization showed up regulation at 24 hpi, the late term after viral infection. In addition, DEGs related to metabolism and histone modification, like Ezh2 were detected, which might correlate with the trained immunity of macrophages. The current in vitro viral infection study showed the key innated immunity character of macrophages, which suggested the replacement value of viral infection cells model, to reduce the animal usage in preclinical researches.

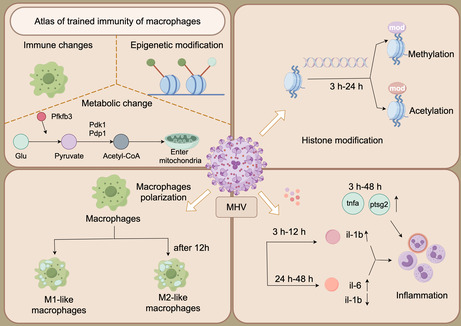

## 1 INTRODUCTION

Coronaviruses are a diverse group of viruses infecting many different hosts. They include SARS‐CoV2, which caused the recent epidemic, middle east respiratory syndrome coronavirus (MERS‐CoV) and SARS‐CoV in human, cattle and bats, and mouse hepatitis virus (MHV) in mice.^1^, ^2^ These highly pathogenic coronaviruses can cause severe acute respiratory diseases, and better understanding of the pathogenesis of viral infection and the immune responses of hosts, and rapid development of vaccines and medicine, and viral infection animal models are urgently needed.^3^ In this in vivo study, the key antiviral roles of macrophages were examined. As major producers of cytokines, macrophages are implicated in inflammatory disease pathogenesis, and orchestrate both innate and adaptive immune responses.^4^, ^5^ In response to cytokines or microbial products present in the microenvironment, macrophages can change their phenotype and become polarized to form M1 or M2 macrophages. M1 macrophages are the classically activated macrophages and are induced by microbial stimuli, producing proinflammatory cytokines. M1 macrophages exhibit proinflammatory and microbicidal properties, and promote extracellular matrix degradation and tissue injury.^6^ The alternative form of macrophages, the M2 macrophages, produce transforming growth factor beta (TGFβ) and Il10, which are involved in the resolution process of the inflammatory response.^7^ M1 and M2 macrophages play important roles in regulating inflammation together.

Mouse hepatitis virus (MHV) is the most common coronavirus in laboratory mouse populations. Raw264.7, a macrophage cell line derived from laboratory mice and the MHV virus were chosen to build a cell model of coronavirus infection as an additional resource for research into pathological mechanisms and drug screening. In this study, transcriptome sequencing and quantitative real‐time PCR (qPCR) were utilized to analyze the expression level of important physiological genes at different time points after infection. Then the feasibility of replacing animal experiments with cell infection models was analyzed by comparing the coronavirus responses reported in the literature.

## 2 METHODS

### 2.1 Cell culture and treatment

Raw264.7 Cells obtained from ATCC were cultured in DMEM medium with 10% fetal bovine serum (FBS) at 37°C with 5% CO_2_. Before viral infection, the medium was changed to DMEM with 1% FBS. 800 TCID_50_ were added per well. At 3, 6, 12, 24, and 48 h post infection (hpi), the culture medium was discarded, and the attached cells were washed with PBS and harvested in Trizol reagent.

### 2.2 Transcriptome sequencing

The samples were sent to Novo Zhiyuan Co., Ltd (Beijing, China) for RNA extraction following the manufacturer's instructions. An analysis of RNA integrity and total amounts was performed using the Bioanalyzer 2100 and the RNA Nano 6000 Assay Kit (Agilent Technologies, Santa Clara, CA, USA). A random hexamer primer and M‐MuLV Reverse Transcriptase were used to synthesize first strand DNA. For selecting cDNA fragments of 370–420 bp in length, the library fragments were purified with the AMPure XP system (Beckman Coulter, Danvers, MA, USA). Following PCR amplification, AMPure XP beads were used to purify the PCR product and generate the library. The quality of the library was ensured by using the Qubit 2.0 fluorometer, the Agilent 2100 bioanalyzer and qRT‐PCR. The different libraries were pooling according to the effective concentration and the target amount of data off the machine. The cDNA fragments were then sequenced using the Illumina NovaSeq 6000. An end reading of 150 bp pairing was generated.

The raw data and the alignment detail can be accessed via GEO accession GSE252673.

### 2.3 RNA‐sequencing data analysis

The read numbers mapped to each gene were count by FeatureCounts (v1.5.0‐p3). Fragments per kilobase (FPKM) were calculated based on a gene's length and read count. Differential gene expression analysis was conducted on R using the DESeq2 R package (1.20.0). The resulting *p* values were adjusted using Benjamini and Hochberg's approach to control the false discovery rate. The threshold for significantly differential expression was established as *p*_adj_ ≤ 0.05 and |log2(foldchange)| ≥ 1.

We applied Gene Ontology (GO) enrichment analysis and KEGG pathways enrichment analysis to differentially expressed genes using the clusterProfiler R package (3.8.1), which corrected gene length bias. KEGG pathways and GO terms with corrected *p* values of less than 0.05 were considered significantly enriched by differential expressed genes.

GSEA reveals key pathways and biological processes in different biological states by enrichment analysis of gene collections on gene expression data. The Hallmark pathway is widely used in GSEA as a carefully selected set of genes, including important genes associated with specific biological states. We combined GSEA with the Hallmark pathway to perform in‐depth analysis of gene expression data at 5 time points to reveal the biological status of macrophages at different times after coronavirus infection. GSEA analysis was performed using the OmicShare tools, a free online platform for data analysis (https://www.omicshare.com/tools).

The protein–protein interaction (PPI) network was constructed using the STRING database (https://string‐db.org/). ‘CytoHubba’ was performed to calculate the weight of each gene and obtain the hub genes of the PPI network. The tool ‘MCODE’ was utilized to identify the representative modules using Cytoscape software 3.8.0.

### 2.4 RNA extraction, reverse transcription, and quantitative real‐time PCR (qPCR)

Total RNA in cells was extracted using the standard RNA extraction method with TRIzol (Invitrogen Life Technologies, USA). After RNA concentration was determined with a NanoDrop spectrophotometer determined, reverse transcription was performed on 1 μg RNA, using the Prime Script RT Reagent Kit [Takara Biomedical Technology (Beijing) Co., Ltd, Cat: RR037A, Beijing, China] to synthesize the cDNA strand, and gene expression was analyzed using an Applied Biosystems® 7500 machine (Life Technologies, Thermo Fisher Scientific, Waltham, MA, USA) with the TB Green® Premix Ex Taq™ II (Tli RNaseH Plus) [Takara Biomedical Technology (Beijing) Co., Ltd, Cat: RR082A]. The primers are shown in Table 1.

**TABLE 1:** Primer sequence for RT‐PCR.

Gene	Forward(5′‐3′)	Reverse(5′‐3′)
Gapdh	CCAATGTGTCCGTCGTGGAT	TGCTGTTGAAGTCGCAGGAG
Cox‐2	CAGGCTGAACTTCGAAACA	GCTCACGAGGCCACTGATACCTA
IL‐6	GCCCACCAAGAACGATAGTCAA	CATTTCCACGATTTCCCAGA
Tnf‐a	GTGCCTATGTCTCAGCCTC	TTGTGAGTGTGAGGGTCTGG
Il‐1β	TGGTACATCAGCACCTCACA	GAAGGCATTAGAAACAGTCC
Pfkfb3	GATCTGGGTGCCCGTCGATCACCG	CAGTTGAGGTAGCGAGTCAGCTTC
Pdk1	AACTGGCCACTTCCAGAGAA	AAAGAAGGGGTGATCCAGGC
Ezh2	TGCCTCCTGAATGTACTCCAA	AGGGATGTAGGAAGCAGTCATAC
Suz12	AGCAACATGGGAGACAATTCTTG	ACAGCAATAGTTTGTGCAGGTTT

### 2.5 Statistical analysis

Statistical analysis was performed with GraphPad Prism (Version 9.5.1) and are expressed as means ± SEM. The experimental data between two groups at different times was compared using the multiple unpaired *t* test. A *p* value < 0.05 was set as the criterion for statistical significance. DESeq2 analysis was used to identify all representative genes differentially expressed between normal cultured cells and MHV‐infected Raw264.7 cells at 3, 6, 12, 24, and 48 h post infection with adjusted values of *p* < 0.05 and log 2FC >0.5.

## 3 RESULTS

### 3.1 Distinct transcriptomic features of Raw264.7 cells post MHV infection

**FIGURE 1:**
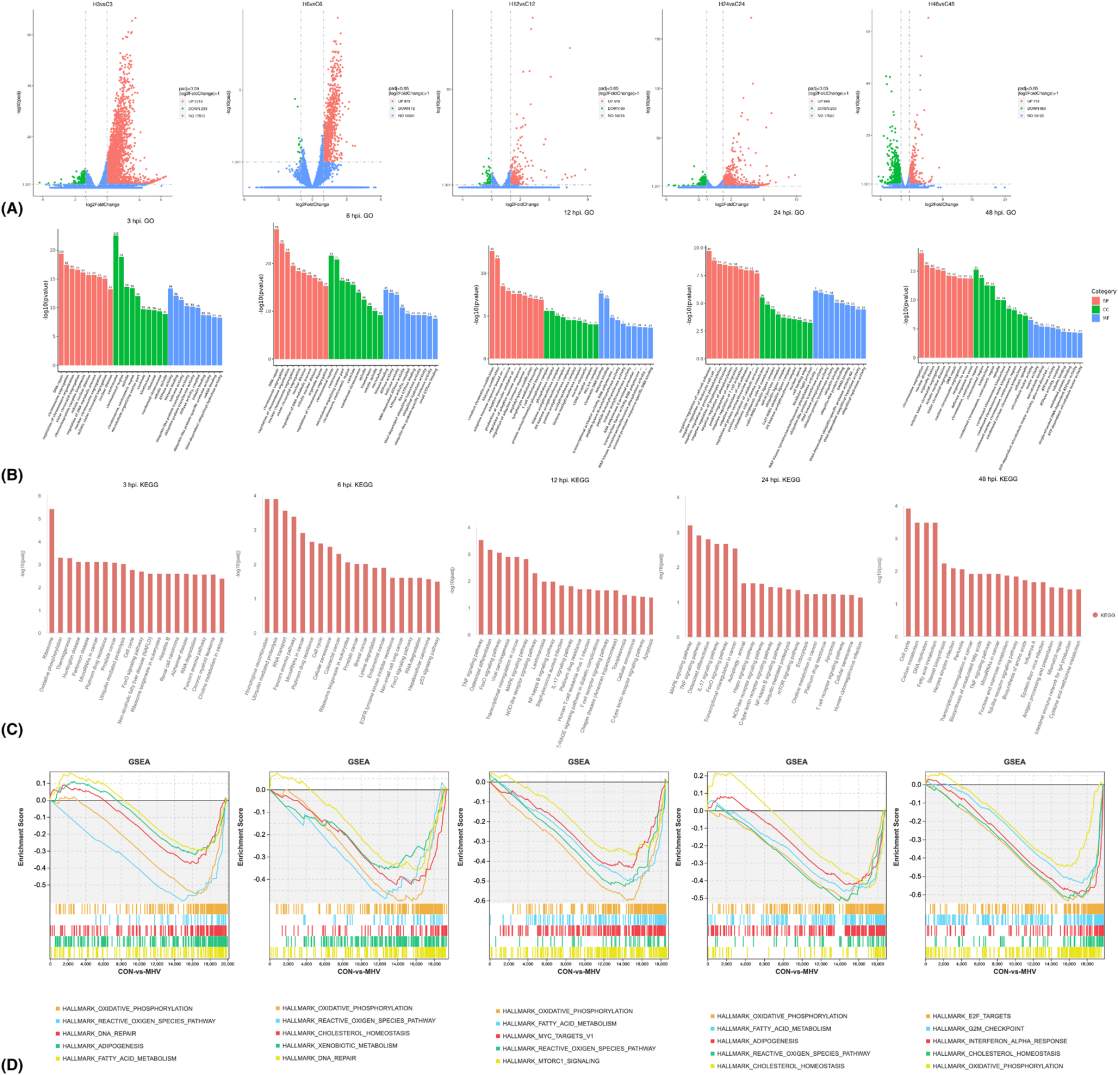
RAW264.7 cell transcriptomic feature changes after MHV viral infection. (A) Volcano plots comparing normal cultured and MHV‐infected Raw264.7 Cells at 3, 6, 12, 24, and 48 h post infection. Green dots and red dots represented the downregulated and upregulated genes, respectively. Blue dots represented genes not significantly changed. (B) Gene ontology (GO) enrichment analysis demonstrated the function of DEGs at different time points (*n* = 3 independent cultures). (C) KEGG enrichment analysis demonstrated the function of DEGs at different time points. (D) GSEA demonstrated the function of DEGs at different time points.

Nearly 19 000 genes were tested. We identified the differentially expressed genes and analyzed their functions through GO analysis. Compared with the uninfected control cells, MHV viral infected cells showed significant differential transcriptomic features (Figure 1). The differentially expressed genes (DEGs) at different time points are shown in the Volcano plots (Figure 1A): 2213 DEGs were upregulated and 293 DEGs were downregulated at 3 hpi; 873 DEGs were upregulated and 12 DEGs were downregulated at 6 hpi; 515 DEGs were upregulated and 90 DEGs were downregulated at 12 hpi; 949 DEGs were upregulated and 250 DEGs were downregulated at 24 hpi; 714 DEGs were upregulated and 989 DEGs were downregulated at 48 hpi. A significant number of DEGs were detected at an early time after infection.

Gene ontology (GO) enrichment analyses were also performed on the DEGs at different time points (Figure 1B). Enrichment of GO terms relevant to cell proliferation, including nuclear division, mitotic sister chromatid segregation, regulation of DNA metabolic process, mitotic spindle, centrosome, and helicase activity, was found at 3 and 6 hpi. At 12 hpi, enrichment of GO terms relevant to immune response and histone modification were found, including adaptive immune response, production of molecular of immune response, regulation of adaptive immune response, histone acetyltransferase complex, H4 histone acetyltransferase complex, protein acetyltransferase complex, and histone methyltransferase complex, while at 24 hpi, some negative regulation of immune response and histone modification GO terms were found, such as negative regulation of cell–cell adhesion, negative regulation of leukocyte cell–cell adhesion, negative regulation of protein phosphorylation, negative regulation of lymphocyte activation, H4 histone acetyltransferase complex and positive regulation of catabolic process. At 48 hpi, GO terms related to antiviral responses, and at the same time GO terms related to cell proliferation were rediscovered. The results of GO enrichment analysis showed that the infected Raw264.7macrophages tended to proliferate in the early stages (3–6 hpi). In the middle stages (12–24 hpi), the related epigenetic regulation occurred, and the immune response was initiated. Finally, the cell proliferation and antiviral responses were again shown in the late stage (48 hpi).

KEGG results (Figure 1C) showed that within 3–6 h after infection, DGEs were mainly enriched in signaling pathways related to cell proliferation, ribosome generation, RNA transport, and ubiquitin mediated protein hydrolysis. In addition, after 6 h, DEGs were also enriched in the Fox3 pathway related to cellular physiological activities, including apoptosis, cell cycle regulation, glucose metabolism, and oxidative stress resistance. This indicates that in the early stages of infection, Raw264.7 cells mainly undergo cell division and begin to regulate intracellular metabolic responses. After 12–24 h, DGEs mainly enrich metabolic related Fox3 pathways, as well as cellular inflammatory responses such as TNF signaling pathway, NOD like receptor signaling pathway, IL‐17 signaling pathway, AGE‐RAGE signaling pathway, T cell receptor signaling pathway, C‐type lectin receptor signaling pathway, cell apoptosis, etc. This indicates that the cells have already begun an inflammatory response at this stage, which mainly relies on the NF‐ κ B signaling pathway, the MAPK signaling pathway, etc. After 48 h, the enriched signaling pathways of DEGs reappeared, along with signaling pathways related to cell cycle and proliferation, as well as Toll like receptor signaling pathways related to the inflammatory response. The results of KEGG are similar to those of GO in terms of cell cycle. At the same time, they further reflect that the cells begin to exhibit inflammatory reactions 12 h after infection, but the mechanisms of inducing and regulating inflammation are not the same in the middle (12 hpi) and late stages (48 hpi) of infection. In the middle stage of infection, the inflammatory response mainly depends on the TNF signaling pathway, the NOD like receptor signaling pathway, the IL‐17 signaling pathway, the AGE‐RAGE signaling pathway, and may also be related to the Toll like receptor signaling pathway in the later stage. In addition, in the KEGG results, multiple genes related to leukemia and cancer were found, possibly due to the source of Raw264.7 cells, which were established from an ascites of a tumor induced in a male mouse by intraperitoneal injection of Abelson Leukemia Virus (A‐MuLV).

The results of GSEA (Figure 1D) showed that cells were mainly engaged in metabolic activities such as oxidative phosphorylation, reactive oxygen species production, fatty acid metabolism and DNA repair within 48 h post infection. In addition, signaling pathways related to cell proliferation, like MYC_TARGETS_V1, E2F_TARGETS, and G2M checkpoint, appeared in the middle to late stages of infection (12 hpi to 48 hpi).

### 3.2 DEGs relevant to inflammation were detected in Raw264.7 macrophages post viral infection

**FIGURE 2:**
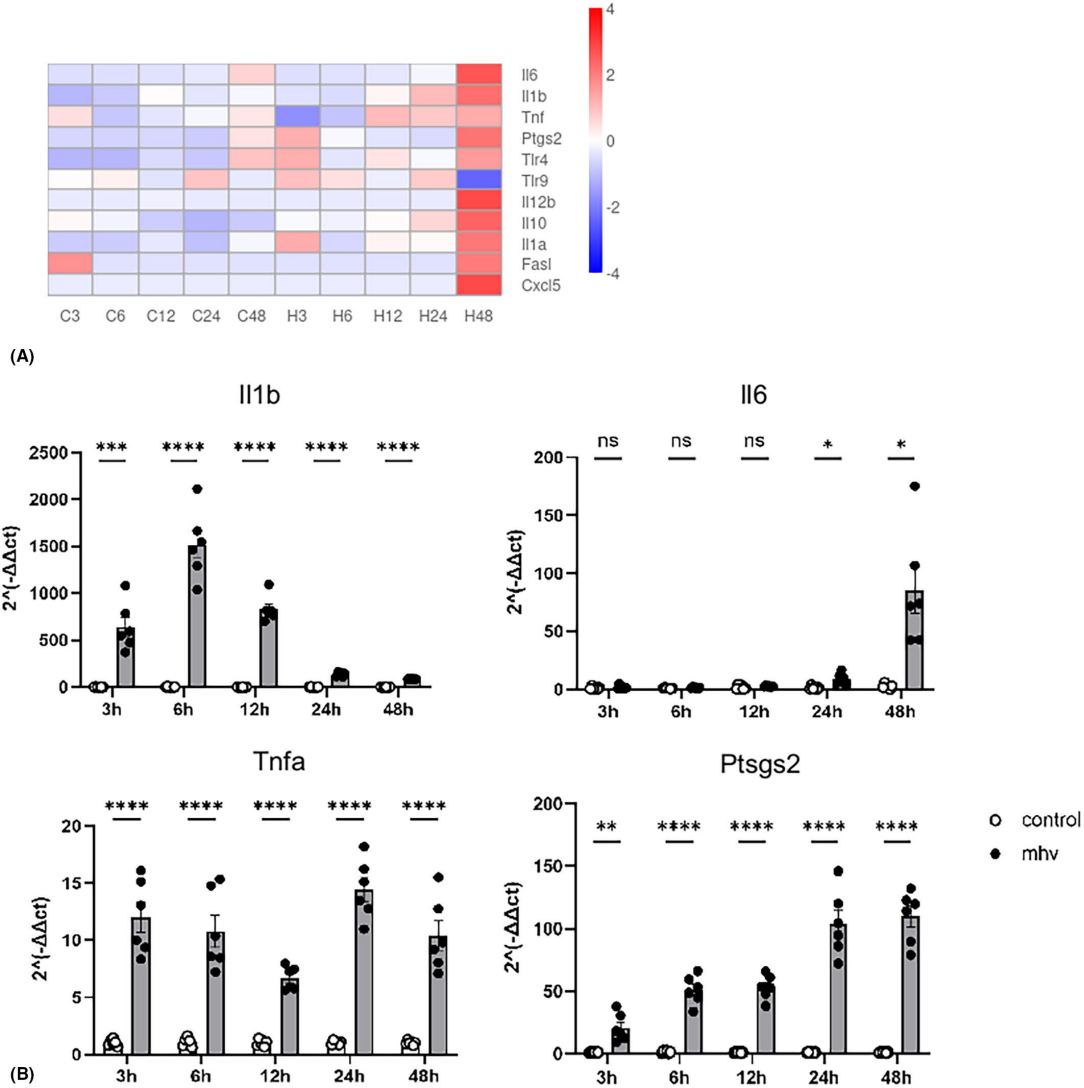
DEGs associated with the inflammation response detected after infection. (A) Heatmap of DEGs related to immune processes in Raw264.7 cells, comparing normal and MHV‐infected Raw264.7 cells at 3, 6, 12, 24, and 48 h. Colors indicate −log10 (*p* value). Relative mRNA levels of Il1b (B), Tnfa (D) and Ptgs2 (E), showing increasing expression levels in the infection group from 3 h to 48 h. (C) Relative mRNA level of Il6, showing a higher expression level in the infection group at 48 h after infection. Data are means ± SEM. *N* = 6 independent cultures in the infected group and the normal group at five time points. *p < 0.05; **p < 0.01; ***p < 0.001; ****p < 0.0001.

After viral exposure, macrophages always act firstly to initiate innate immunity, and a series of gene transcriptions associated with inflammatory response might start. In the current in vitro transcriptome study, the classic inflammatory genes^8^, ^9^ were selected for differential transcription analysis. The heatmap showed upregulation of these inflammatory genes at different time points after viral infection when compared with those uninfected cells, and almost all of the inflammatory genes except *Tlr9* showed significantly elevated transcription at 48 hpi. In the transcriptome data, *Il1b* and *Ptgs2* (encoding cyclooxygenase 2, COX‐2) showed higher elevation at almost all the time points post viral infection, while some other genes like *Il6*, *Il12b*, and *Cxcl5* only showed significant elevation at 48 hpi (Figure 2A).

Fluctuation in gene transcription may lead to the observed variation in the transcriptome data, even though triplicated samples were included at different time points. To confirm the transcriptional changes, the experiments were repeated and RNA samples were collected and analyzed by qPCR (Figure 2B). The RNA of *Il6* and *Ptgs2* detected by qPCR show similar trends to the transcriptome data, although the RNA of *Tnfa* and *Il1b* showed constant elevation post infection, which was not completely consistent with the transcriptome data.

### 3.3 DEGs relevant to macrophage ‘alternative’ polarization and glycolysis elevated at a later infection stage

**FIGURE 3:**
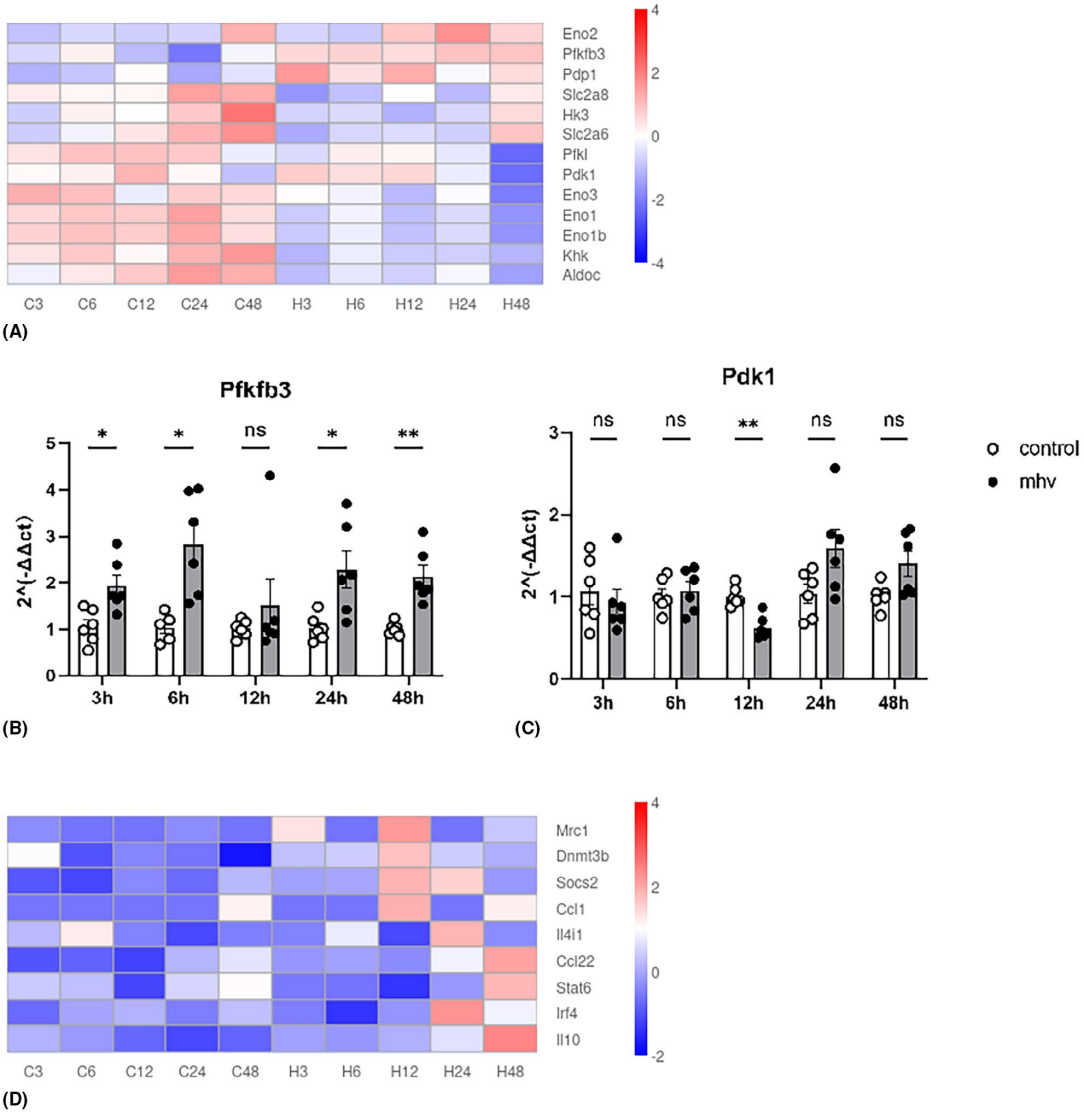
DEGs associated with glycolysis and macrophages polarization after infection. (A) Heatmap of DEGs related to glycolysis in Raw264.7 cells, comparing normal and MHV‐infected Raw264.7 cells at 3, 6, 12, 24, and 48 h. Colors indicate −log10 (*p* value). (B) Relative mRNA levels of Pfkfb3, showing a higher expression level in the infection group at 3, 6, 24, and 48 h after infection. (C) Relative mRNA levels of Pdk1, showing higher levels in the infection group at 24 h. (D) Heatmap of DEGs related to ‘alternative’ anti‐inflammatory M2 Raw264.7 macrophages, comparing normal and MHV‐infected Raw264.7 cells at 3, 6, 12, 24, and 48 h. Colors indicate −log10 (*p* value). Data are means ± SEM. *N* = 5–6 independent cultures in the infected group and the normal group at five time points. *p < 0.05; **p < 0.01; ***p < 0.001; ****p < 0.0001. (D) Heatmap of DEGs related to ‘alternative’ anti‐inflammatory M2 Raw264.7 macrophages, comparing normal and MHV‐infected Raw264.7 cells at 3, 6, 12, 24, and 48 h. Colors indicate −log10 (*p* value).

The antiviral response of macrophage needs a lot of energy. In the current in vitro transcriptome study, the classic genes related to glycolysis^10^, ^11^ were selected for differential transcription analysis. The heatmap shows changes of these metabolism genes at different time points after viral infection compared with those in uninfected cells. The majority of the glycolysis genes showed a significant decline in transcription after infection, but at the same time, the expression of genes that regulate key enzymes like *Pfkfb3* and *Pdk1* in glycolysis were upregulated (Figure 3A). The relative mRNA level of *Pfkfb3* (Figure 3B) tested by qPCR shows a similar trend to the heatmap but the relative mRNA level of *Pdk1* (Figure 3C) decreased at 12 h, which is inconsistent with the transcriptome data.

M2 macrophages produce transforming growth factor beta (TGF‐β) and IL‐10, which are involved in the resolution process of the inflammatory response.^7^ We have observed the expression levels of genes related to inflammation before; the heatmap in Figure 3D shows that the expression of M2 related genes gradually increased 12 h after infection with the virus, indicating that cells began to limit their own inflammatory response at 12 hpi.

These results do not necessarily indicate whether the glycolytic activity of the macrophages in our experiments increased or decreased after infection with MHV because, although most genes are downregulated, the expression of genes that regulate key enzymes in glycolysis were upregulated. However, in our sequencing and qPCR experiments, we observed upregulation of gene expression of *Pfkfb3*, which is related to immune response regulation while also playing a key role in glycolysis,^12^, ^13^, ^14^ indicating that intracellular glycolysis in infected cells undergoes changes with the occurrence of the cellular inflammatory response. Further experiments are needed to demonstrate the changes in cellular metabolism.

### 3.4 DEGs relevant to histone modification prompted macrophage epigenetic regulation in anti‐viral responses

**FIGURE 4:**
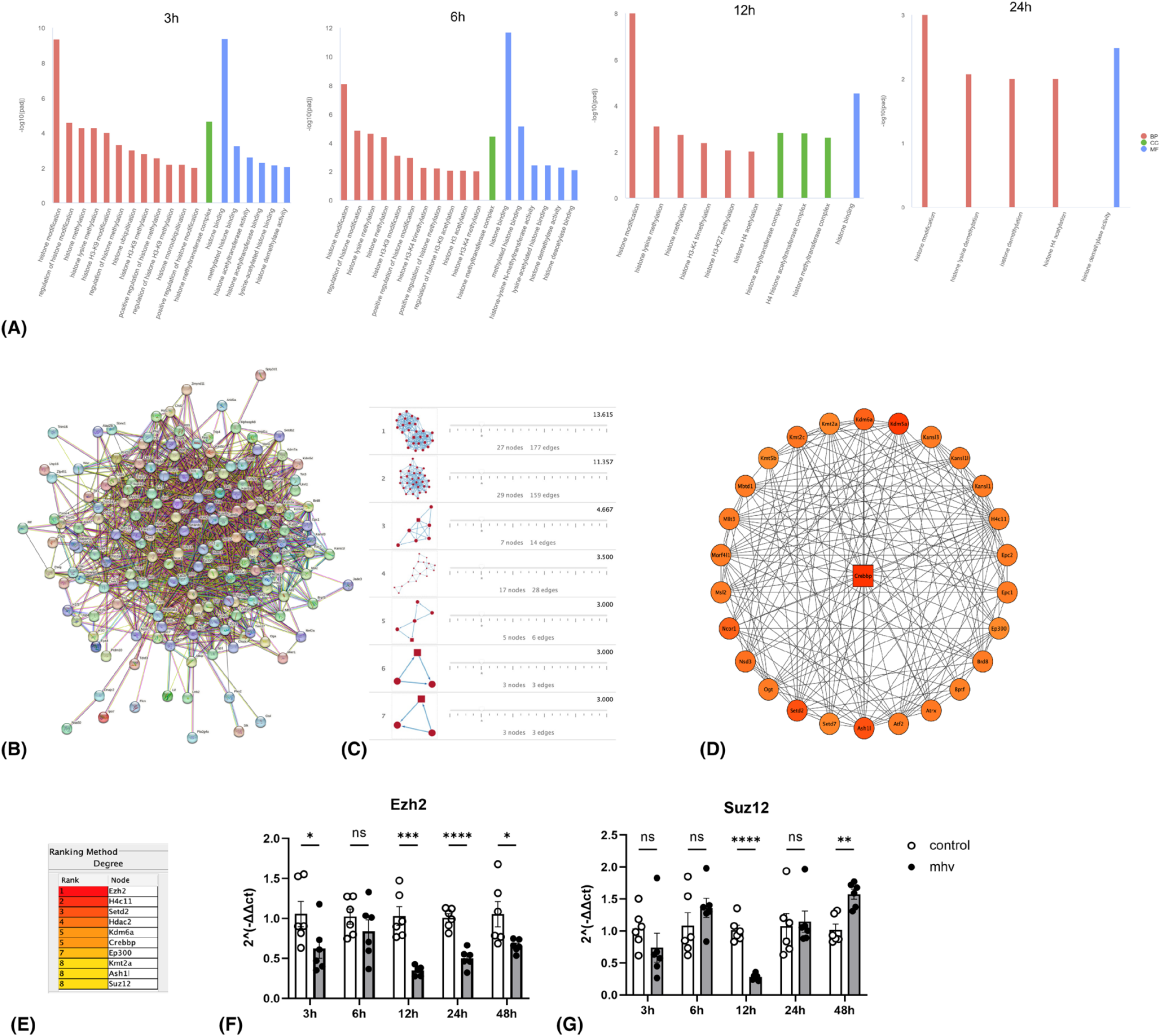
GO terms and content genes related to histone modification. (A) GO terms relevant to histone (keyword contains histone, *p*_adj_ < 0.01) at different times after infection. (B) PPI network of 163 genes associated with histone. (C) A total of 7 modules were obtained from MCODE analysis. (D) Crebbp was screened as the core gene among model1 by MCODE. (E) The top 10 connectivity nodes in model1 analyzed by cytohubba. (F) Relative mRNA levels of Ezh2, showing a lower expression level in the infection group at 3, 12, 24, and 48 h after infection. (G) Relative mRNA levels of Suz12, showing lower levels early in infection group at 12 h and higher levels at 48 h. Data are means ± SEM. *N* = 6 independent cultures in the infected group and the normal group at five time points. *p < 0.05; **p < 0.01; ***p < 0.001; ****p < 0.0001.

Epigenetics regulates the expression of DNA‐encoded information and determines the specific ‘identity’ of a cell. It has been revealed that developmental or environmental signals can dynamically alter epigenetic chromatin markers.^15^ Epigenetic changes can be divided into three categories: (1) posttranscriptional histone modifications, (2) DNA methylation, and (3) noncoding RNA.^16^ In our GO analysis of DEGs, some terms related to histone modification were observed at 12 h and 24 h after infection, and important cellular physiological changes like inflammatory response and M2 polarization were also observed. This implies that histone modification plays a critical role in cellular physiological changes. GO terms related to histone (keyword contains histone, *p*_adj_ < 0.01) were selected (Figure 4A) and methylation and acetylation were observed on H3 and H4 at 3 to 24 hpi; no eligible GO term was obtained at 48 hpi. Then 163 genes from these GO terms (genes are listed in the accompanying documents) were collected for protein–protein interaction (PPI) network analysis to find the key gene in histone modification which induced the cellular physiological changes (Figure 4B). ‘MCODE’ was used to identify the representative modules. A total of 7 modules were obtained from MCODE analysis (Figure 4C), and *Crebbp* was screened as the core gene among model1 (Figure 4D). The top 10 connectivity nodes among the 163 genes were found by cytohubba, including *Ezh2*, *Setd2*, and *Suz12*, and also *Crebbp* found by MCODE (Figure 4E). The relative mRNA levels of *Ezh2* (Figure 4F) and *Suz12* (Figure 4G) showed different expression levels in the infection group. The results show that these 10 genes may play important roles in histone modification, which induces the cellular physiological changes.

## 4 DISCUSSION

MHV strains vary in virulence, organotropism, and cellularity, and have evolved through naturally occurring mutations and recombination, becoming the prototype for studying the molecular biology of coronavirus.^17^, ^18^ In this study, we established MHV‐infected Raw264.7 cell model and found, using transcriptome sequencing and qPCR, that the expression level of genes related to important physiological processes like inflammation, glycolysis, M2 polarization and epigenetic modification changed at different time points after infection.

Firstly, we found that different inflammatory genes changed during coronavirus infection. The expression of some genes such as *Il1b* increased significantly in the early stages of infection (3–12 hpi) and the degree of gene expression upregulation decreased in the later stages (24–48 hpi). Some genes, like *Il6*, were more highly expressed only in the late stage. This suggested that *Il1b* and *Il6* played an important role in inducing inflammation and cascade enhancement of inflammatory response. Genes that were upregulated at all stages after infection, like *Tnfa* and *Ptgs2*, may be related to the maintenance of infectious inflammation. In other clinical and in vivo studies, IL‐1 and IL‐6 were found played an important role in the cytokine storm^19^, ^20^ and were the main factors leading to tissue injury. Anti‐IL‐1 or anti‐IL‐6 therapy have proved effective in infectious inflammation.^21^, ^22^, ^23^

Furthermore, genes related to inflammation are also associated M1 macrophages. These are the classically activated macrophages which exhibit proinflammatory and microbicidal properties, and promote extracellular matrix degradation and tissue injury. Acute inflammatory activation of macrophages must be transient and tightly regulated to prevent tissue damage.^6^ Tolerance follows the activation state, which is inherently unstable. Alternatively activated M2 macrophages release anti‐inflammatory cytokines and decrease the production of proinflammatory cytokines. They also show a decreased ability to bind antigens. M2 macrophages are involved in the resolution process of the inflammatory response and wound healing by extracellular matrix construction, cell proliferation, angiogenesis, and stimulation of collagen production.^7^ Interestingly, we found that genes related to M2 were highly expressed from 12 h after MHV infection. Taken together with previous results, we found that after coronavirus MHV infection, the majority of inflammatory genes showed a continuous high expression level, with genes related to M2 expression upregulating in the late infection stage (12–48 h). This indicates that macrophages are hyperactivated. This phenomenon has been reported in many clinical studies and animal experiments. Macrophages in patients with severe COVID‐19 become overactivated and cause a storm of inflammatory cytokines, dominated by IL‐6.^24^ Our cell model mimicked this process well in vitro and the relevant mechanism needs further study.

In the term of metabolism, our results do not necessarily indicate whether the glycolytic activity of macrophages in the experiments increases or decreases after infection with MHV. Although most genes are downregulated, the expression of genes that regulate key enzymes in glycolysis is upregulated. We observed upregulation of gene expression of *Pfkfb3*, which is related to immune response regulation while also playing a key role in glycolysis,^12^, ^13^, ^14^ indicating that intracellular glycolysis in infected cells undergoes changes with the occurrence of a cellular inflammatory response. Further experiments are needed to demonstrate the changes in cellular metabolism.

The anti‐virus processes of macrophages have to be highly effective at secreting cytokines, recruiting innate immune cells, phagocytosis, and killing of pathogens or cells, all of which require high energy.^25^ Glycolysis is a major metabolic pathway and plays an important role in biosynthetic pathways. The ‘Warburg Effect’, seen in cancer cells, tends to ‘ferment’ glucose into lactate, even in the presence of sufficient oxygen to support mitochondrial oxidative phosphorylation,^26^ which has been identified as a key metabolic transition to glycolysis. It has been seen in cells undergoing biosynthesis of pro‐inflammatory molecules.^27^, ^28^, ^29^

In terms of epigenetic modification, we noticed histone modification in GO analysis of DEGs. We found repressive histone modification markers H3K9 methylation and H3K27 acetylation and active histone modification markers H3K4 methylation.^12^ This suggests that these epigenetic modification mechanisms play a central role in the changes to macrophage physiological activities during coronavirus infection. In addition, we also noticed the immune memory ability of macrophages. The histone modification and changes in glycolysis we observed before are important mechanisms of macrophage memory. The formation of repressive histone modification markers H3K9 methylation and H3K27 acetylation enables macrophages to develop tolerance^12^, ^16^ and they may not produce or produce only a weak immune response in the face of the next pathogenic infection. This may be one of the reasons for the increase in bone marrow‐derived suppressor cells in patients with COVID‐19 infection reported in some studies.^30^

The emergence of SARS‐CoV‐2 led to the largest global pandemic in living memory, with between 4.5 and 15 M deaths globally from COVID‐19. A consistent feature of severe COVID‐19 is dysregulation of pulmonary macrophages, cells that under normal physiological conditions play vital roles in maintaining lung homeostasis and immunity. SARS‐CoV‐2‐infected AMs produce higher levels of pro‐inflammatory IL1B cytokines than their uninfected counterparts. Clinical studies have found that IL1 and IL6 secreted by macrophages can cause severe lung inflammation. Dexamethasone inhibits the secretion of inflammatory factors by macrophages and can significantly reduce mortality. Loss of resident AMs may occur as a collateral result of hyper‐inflammation. Understanding the pathogenesis of inflammation‐driven diseases remains critical to understanding how best to intervene with therapeutic agents in clinical trials and has been a major focus of COVID‐19 research.^31^

In addition, M1 and M2 macrophages have always been considered as two separate states of the macrophage inflammatory response, namely promoting inflammation and regulating and inhibiting the inflammatory response. Recent transcriptional profiling of macrophages shows that tissue resident macrophages can have both ‘M1’ and ‘M2’ features.^32^, ^33^

## 5 CONCLUSIONS

In summary, these in vitro transcriptome data simulated well the innate immune response of macrophages after viral infection in vivo and show that this method of in vitro study may be used to complement or replace in vitro components of laboratory animal research into viral infection.

## AUTHOR CONTRIBUTIONS

**Yun Liu**: Conceptualization, Methodology, Formal analysis, Investigation, Resources, Data Curation, Writing ‐ Original Draft, Visualization. **Ting‐Ting Feng**: Formal analysis, Investigation, Resources, Visualization, Data curation. **Wei Tong**: Investigation. **Zhi Guo**: Investigation. **Xia Li**: Investigation. **Qi Kong**: Formal analysis. **Zhi‐Guang Xiang**: Project administration, Supervision, Funding acquisition, Validation, Writing ‐ Review & Editing.

## FUNDING INFORMATION

This work was supported by the CAMs innovation Fund for Medical Sciences (2022‐12M‐CoV19‐005) and grant of National Key Projects (2023YFF0724900 and 2021YFF0702802).

## CONFLICT OF INTEREST STATEMENT

The authors declare no conflict of interest in this study.

## ETHICS STATEMENT

None of the experiments and procedures on animals is contained in this study.

## References

[1] Cui J , Li F , Shi Z‐L . Origin and evolution of pathogenic coronaviruses. Nat Rev Microbiol. 2019;17:181‐192. 30531947 10.1038/s41579-018-0118-9 PMC7097006

[2] Wu JT , Leung K , Leung GM . Nowcasting and forecasting the potential domestic and international spread of the 2019‐nCoV outbreak originating in Wuhan, China: a modelling study. Lancet. 2020;395:689‐697. 32014114 10.1016/S0140-6736(20)30260-9 PMC7159271

[3] Fan C , Wu Y , Rui X , et al. Animal models for COVID‐19: advances, gaps and perspectives. Signal Transduct Target Ther. 2022;7:220. 35798699 10.1038/s41392-022-01087-8 PMC9261903

[4] Wynn TA , Chawla A , Pollard JW . Origins and hallmarks of macrophages: development, homeostasis, and disease. Nature. 2013;496:445‐455. 23619691 10.1038/nature12034 PMC3725458

[5] Chu Z , Sun C , Sun L , et al. Primed macrophages directly and specifically reject allografts. Cell Mol Immunol. 2020;17:237‐246. 30948792 10.1038/s41423-019-0226-0 PMC7052205

[6] Ivashkiv LB . Inflammatory signaling in macrophages: transitions from acute to tolerant and alternative activation states. Eur J Immunol. 2011;41:2477‐2481. 21952800 10.1002/eji.201141783 PMC3264328

[7] Mosser DM , Edwards JP . Exploring the full spectrum of macrophage activation. Nat Rev Immunol. 2008;8:958‐969. 19029990 10.1038/nri2448 PMC2724991

[8] Jeyanathan M , Vaseghi‐Shanjani M , Afkhami S , et al. Parenteral BCG vaccine induces lung‐resident memory macrophages and trained immunity via the gut–lung axis. Nat Immunol. 2022;23:1687‐1702. 36456739 10.1038/s41590-022-01354-4 PMC9747617

[9] Ortiz Peña N , Cherukula K , Even B , et al. Resolution of MoS2 nanosheets‐induced pulmonary inflammation driven by nanoscale intracellular transformation and extracellular‐vesicle shuttles. Adv Mater. 2023;35:e2305230. 37534384 10.1002/adma.202305230

[10] Jaroonwitchawan T , Arimochi H , Sasaki Y , et al. Stimulation of the farnesoid X receptor promotes M2 macrophage polarization. Front Immunol. 2023;14:1065790. 36776885 10.3389/fimmu.2023.1065790 PMC9911659

[11] Huangfu N , Zheng W , Xu Z , et al. RBM4 regulates M1 macrophages polarization through targeting STAT1‐mediated glycolysis. Int Immunopharmacol. 2020;83:106432. 32248017 10.1016/j.intimp.2020.106432

[12] Drummer C , Saaoud F , Shao Y , et al. Trained immunity and reactivity of macrophages and endothelial cells. Arterioscler Thromb Vasc Biol. 2021;41:1032‐1046. 33380171 10.1161/ATVBAHA.120.315452 PMC7904591

[13] Keating ST , Groh L , Thiem K , et al. Rewiring of glucose metabolism defines trained immunity induced by oxidized low‐density lipoprotein. J Mol Med Berl Ger. 2020;98:819‐831. 10.1007/s00109-020-01915-w PMC7297856 32350546

[14] Zhang J , Muri J , Fitzgerald G , et al. Endothelial lactate controls muscle regeneration from ischemia by inducing M2‐like macrophage polarization. Cell Metab. 2020;31:1136‐1153.e7. 32492393 10.1016/j.cmet.2020.05.004 PMC7267778

[15] Davidson EJ , Yang IV . Role of epigenetics in the development of childhood asthma. Curr Opin Allergy Clin Immunol. 2018;18:132‐138. 29389731 10.1097/ACI.0000000000000429 PMC8169082

[16] Chen S , Yang J , Wei Y , Wei X . Epigenetic regulation of macrophages: from homeostasis maintenance to host defense. Cell Mol Immunol. 2020;17:36‐49. 31664225 10.1038/s41423-019-0315-0 PMC6952359

[17] Homberger FR . Enterotropic mouse hepatitis virus. Lab Anim. 1997;31:97‐115. 9175007 10.1258/002367797780600189

[18] Haring J , Perlman S . Mouse hepatitis virus. Curr Opin Microbiol. 2001;4:462‐466. 11495812 10.1016/S1369-5274(00)00236-8 PMC7129732

[19] Durand M , Troyanov Y , Laflamme P , Gregoire G . Macrophage activation syndrome treated with anakinra. J Rheumatol. 2010;37:879‐880. 20360206 10.3899/jrheum.091046

[20] Teachey DT , Rheingold SR , Maude SL , et al. Cytokine release syndrome after blinatumomab treatment related to abnormal macrophage activation and ameliorated with cytokine‐directed therapy. Blood. 2013;121:5154‐5157. 23678006 10.1182/blood-2013-02-485623 PMC4123427

[21] van der Stegen SJC , Davies DM , Wilkie S , et al. Preclinical *in vivo* modeling of cytokine release syndrome induced by ErbB‐retargeted human T cells: identifying a window of therapeutic opportunity? J Immunol. 2013;191:4589‐4598. 24062490 10.4049/jimmunol.1301523

[22] Kang S , Tanaka T , Narazaki M , Kishimoto T . Targeting Interleukin‐6 signaling in clinic. Immunity. 2019;50:1007‐1023. 30995492 10.1016/j.immuni.2019.03.026

[23] Fajgenbaum DC , June CH . Cytokine Storm. N Engl J Med. 2020;383:2255‐2273. 33264547 10.1056/NEJMra2026131 PMC7727315

[24] McGonagle D , Sharif K , O'Regan A , Bridgewood C . The role of cytokines including interleukin‐6 in COVID‐19 induced pneumonia and macrophage activation syndrome‐like disease. Autoimmun Rev. 2020;19:102537. 32251717 10.1016/j.autrev.2020.102537 PMC7195002

[25] Netea MG , Domínguez‐Andrés J , Barreiro LB , et al. Defining trained immunity and its role in health and disease. Nat Rev Immunol. 2020;20:375‐388. 32132681 10.1038/s41577-020-0285-6 PMC7186935

[26] Vander Heiden MG , Cantley LC , Thompson CB . Understanding the Warburg effect: the metabolic requirements of cell proliferation. Science. 2009;324:1029‐1033. 19460998 10.1126/science.1160809 PMC2849637

[27] Kleinnijenhuis J , Quintin J , Preijers F , et al. BCG‐induced trained immunity in NK cells: role for non‐specific protection to infection. Clin Immunol. 2014;155:213‐219. 25451159 10.1016/j.clim.2014.10.005 PMC5084088

[28] Jha AK , Huang SCC , Sergushichev A , et al. Network integration of parallel metabolic and transcriptional data reveals metabolic modules that regulate macrophage polarization. Immunity. 2015;42:419‐430. 25786174 10.1016/j.immuni.2015.02.005

[29] Donnelly RP , Finlay DK . Glucose, glycolysis and lymphocyte responses. Mol Immunol. 2015;68:513‐519. 26260211 10.1016/j.molimm.2015.07.034

[30] Alsalman A , Al‐Mterin MA , Elkord E . Role of T regulatory cells and myeloid‐derived suppressor cells in COVID‐19. J Immunol Res. 2022;2022:1‐13. 10.1155/2022/5545319 PMC9042623 35497875

[31] Bain CC , Rossi AG , Lucas CD . Pulmonary macrophages and SARS‐Cov2 infection. Int Rev Cell Mol Biol. 2022;367:1‐28. 35461655 10.1016/bs.ircmb.2022.01.001 PMC8968207

[32] Gibbings SL , Thomas SM , Atif SM , et al. Three unique interstitial macrophages in the murine lung at steady state. Am J Respir Cell Mol Biol. 2017;57:66‐76. 28257233 10.1165/rcmb.2016-0361OC PMC5516280

[33] Lavin Y , Winter D , Blecher‐Gonen R , et al. Tissue‐resident macrophage enhancer landscapes are shaped by the local microenvironment. Cell. 2014;159:1312‐1326. 25480296 10.1016/j.cell.2014.11.018 PMC4437213

